# A case of leprosy in Malawi. Making the final push towards eradication: a clinical and public health perspective

**DOI:** 10.1186/s40249-016-0176-z

**Published:** 2016-09-02

**Authors:** Cieron Roe, Lucy Sarah May

**Affiliations:** Brighton and Sussex Medical School, Brighton, UK

**Keywords:** Leprosy, Eradication, Africa, World Health Organisation, Malawi, International complication, Communicable disease, Tropical

## Abstract

Statistically speaking, Malawi has achieved the World Health Organisation's target for the elimination of leprosy (<1 case per 10 000 people), yet the disease is still considered a leading cause of long term physical disability. In this case study the authors discuss the presentation of a 39-year-old gentleman to a district hospital in Malawi with multibacillary, lepromatous leprosy. The condition was initially managed in the community as an ‘allergy’ which suggests that local barriers currently hinder the detection of leprosy in this developing primary care system. Leprosy is a multi-system disease and this gentleman demonstrated evidence of lepromatous orchitis. Promoting an awareness of these systemic manifestations will increase the the detection of complications and circumvent long term morbidity. Efforts to optimise systems of detection, management and public and professional education are essential to drive eradication in these at-risk populations. At an international level, we must strive to fulfil the objectives outlined by the ‘Enhanced Global Strategy for Further Reducing the Disease Burden due to Leprosy for 2011–2015’. At a national level, local research should delineate community factors that impede the eradication of leprosy. Developing new diagnostic and epidemiologic tools, more efficacious chemoprophylactic regimens and vaccination for endemic regions would facilitate these efforts.

**Electronic supplementary material:**

The online version of this article (doi:10.1186/s40249-016-0176-z) contains supplementary material, which is available to authorized users.

## Multilingual abstracts

Please see Additional file 1 for translations of the abstract into the six official working languages of the United Nations.

## Background

Leprosy has been endemic in many regions of the world since biblical times. In 2014, 213 899 new cases were detected worldwide [1]. *Mycobacterium leprae* (*M. leprae*) is transmitted following prolonged close contact with infected patients. The bacilli reside within upper airway secretions and spread through the expulsion and consequent inhalation of airbourne droplets. Although highly contagious, the clinical disease only develops in genetically susceptible individuals. The quality of the immune response determines the outcome of infection. Ridley and Jopling characterised a spectrum based on clinical, histological and immunological variables. They defined three broad syndromes: tuberculoid, borderline and lepromatous leprosy [2, 3]. The latter develops when CD4^+^ T cells preferentially polarise to a T helper 2 response. This promotes humoral immunity which fails to clear the intracellular bacilli. Leprosy is characterised by a remarkably long incubation period spanning between six months and twenty years [4]. Lepromatous lesions are characterised by deteriorating sensitivity to heat, pain and tactile sensation as the peripheral nerves denervate. Multiple large plaques develop symmetrically over colder areas of the body. They are hypochromic, erythematous and have indefinite borders. Over time, some of these plaques form nodules, known as lepromas. Borderline cases develop hypochromic spots with slight insensitivity whilst tuberculoid leprosy is characterised by a few isolated erythematous plaques.

## Case presentation

**Fig. 1:**
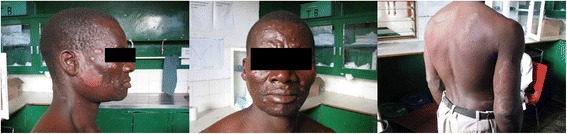
Presentation of widespread skin lesions consistent with lepromatous leprosy. The patient is beginning to develop leonine facies with a large number of papules and nodules afflicting the face, chest, back, legs and groin. Large well circumscribed plaques were noted on the triceps aspect of the right arm and over his left shoulder and right flank

A 39 year old gentleman initially presented to a community health centre in Malawi’s Lilongwe district with widespread skin lesions on the face, torso and the triceps aspect of the right arm. He was referred to the neighbouring district hospital with ‘allergies’. The lesions were long-standing and had evolved relatively slowly. He was otherwise well although concerned about patches of anaesthetic skin on his face and on the soles of his feet. He denied any past medical history although his blood pressure was incidentally raised at 150/93 mmHg. He smoked occasionally and drank moderate amounts of alcohol. On examination the gentleman had widespread skin nodules and scaly plaques with characteristically thick dermis on his cheeks and feet. He had thick facial nodules and there was evidence of eyebrow loss; symptoms consistent with early leonine facies (Fig. 1). This appearance and the description of lesion anaesthesia and peripheral neuropathy were highly suggestive of lepromatous disease. He also had slight discomfort in his right upper quadrant and mild right testicular tenderness and swelling. Due to limited resources, the only suitable laboratory investigations available were a full blood count and urea and electrolytes. The only abnormality was a mildly raised white cell count (12x10^9^/L). The gentleman was assigned a clinical diagnosis of leprosy as defined by the World Health Organisation’s (WHO) standard diagnostic criteria in Table 1 [5]. Consequently, he was referred to Kamuzu Central Hospital for treatment with multidrug chemotherapy (100 mg Dapsone, 50 mg Clofazimine and 600 mg Rifampicin for 12 months).

**Table 1:** The WHO diagnostic features for leprosy

World Health Organisation operational diagnostic criteria for leprosy	
1. Skin lesion consistent with leprosy and with definite sensory loss, with or without thickened nerves	
2. Positive skin smears	

*In an endemic country or area, an individual should be regarded as having leprosy if he or she shown ONE of the above cardinal signs. Only in rare instances is there a need to use laboratory and other investigations to confirm a diagnosis. Adapted from WHO* [5]

## Case discussion

### Clinical perspective

In all settings, a detailed travel, social and contact history can accurately predict the risk of exposure. Moet et al. showed that the most important determinants of clinical disease include i) the intensity of contact and ii) inherited susceptibility including HLA-DR2 and non-HLA polymorphism e.g. in TNF-α [6]. Seropositivity represents a 20-fold increased risk of developing clinical leprosy [7, 8] whilst the bacterial load, as measured by a skin smear can predict the transmission risk [6]. Differential diagnoses for multibacillary lepromatous skin lesions include scleroderma, mycosis fungoides, pellagra, asteatosis, ichtyosis and eczema or contact dermatitis. The primary neurological symptoms resemble common mononeuropathies. Where there is systemic involvement, clinicians should consider systemic lupus erythematosus, rheumatoid arthritis, dermatopolymyositis and systemic vasculitis.

Ideally, leprosy should be confirmed by the presence of acid-fast bacilli in skin biopsies or split skin smears. *M. Leprae* is a gram variable organism and can be decolourised by a Ziehl-Neelsen stain thus a modified Fite stain should be used. Skin biopsies should be taken from the centre of skin plaques or nodules in lepromatous leprosy and the periphery of tuberculoid lesions. Despite being a major referral centre, this district hospital did not have the laboratory facilities to perform split skin smears or skin biopsies. Whilst the WHO standard diagnostic criteria state that these investigations are not required to make a formal diagnosis (Table 1), this situation highlights the inadequacy of laboratory provisions in the Lilongwe health district. To achieve eradication targets, these secondary care institutions require access to reliable, definitive laboratory investigations. Boyce et al. demonstrate that strategies to improve investment at a national and district level are a cost-effective means of increasing detection rates, reducing misdiagnosis and improving the follow up of treatment [9].

This gentleman also complained of a painful and swollen right testicle. The *M. Leprae* bacterium can cause multi-system pathology; approximately 90 % of men with lepromatous leprosy develop orchitis [4]. Table 2 summarises the extent of multi-system involvement. An awareness of these conditions will facilitate early detection and treatment, and prevent the development of long term co-morbidities e.g. infertility.

**Table 2:** Systems with a significant degree of *M. Leprae* infiltration and/or dysfunction

System	Complication(s)	Reference(s)
Renal system	Secondary amyloidosis can develop in several organs although it commonly causes kidney damage by glomerulonephritis (incidence up to 50 %), interstitial nephritis, nephrotic syndrome, pyelonephritis and acute tubular necrosis.	[4, 18]
Respiratory system	*M. leprae* can infiltrate the upper respiratory tract including the nose, pharynx, larynx, epiglottis and trachea. Common symptoms include cough, hoarseness, and, occasionally, dyspnoea. The bronchi and lungs are usually spared.	[18–20]
Cardiovascular system	Leprosy (particularly multibacillary) has been associated with arrhythmias, dyspnoea, ventricular hypertrophy and ST segment abnormalities. *M. Leprae* can invade the cardiac autonomic system. Coronary artery disease and arteriographic abnormalities affect approximately 11 and 50 % of patients respectively.	[4, 18]
Endocrine system	Approximately 90 % of men develop orchitis, commonly with involvement of the epididymis. This can lead to infertility, sexual impotence and gynaecomastia. Approximately 30 % of patients develop cortical adrenal lesions.	[18, 20, 21]
Hepato-biliary system	The incidence of liver involvement is estimated at between 48 % and almost 100 %. Lepromatous hepatitis describes portal and centrolobular granulomas, and fibrosis with acid-fast bacilli. Infrequently, secondary amyloidosis can cause hepatic damage. The gall bladder, biliary system and pancreas are usually spared.	[18, 22]
Haematology	90 % of lepromatous patients show bacillaemia. Bone marrow infiltration can lead to pancytopaenia. A widespread lymphadenopathy can also occur.	[4, 18]
Reproductive system	Approximately 90 % of infected males develop orchitis. Foamy macrophages often form granulomas which replace the testicular parenchyma. Otherwise, parenchymal fibrosis and hyalinization of the spermatopoietic tubules may occur. If left untreated this will eventually cause testicular atrophy and infertility. There is very little involvement of the female genital tract.	[18, 20]

Systemic involvement is fully reviewed by Klioze et al. [18]. Organ dysfunction correlates with: (i) the degree of infiltration; and (ii) interaction with other factors including amyloid infiltration, infection, leprosy reactions and concomitant drug use [18]

### Public health perspective

The WHO’s ‘Strategic Plan for Leprosy Elimination 2000–2005’ made a commitment to eliminate leprosy through control measures that are accessible at all local health facilities [10]. These control measures emphasised facilities to diagnose and treat patients with multidrug chemotherapy, patient and family counselling, community education, disability support, rehabilitation and referrals for complications. In some nations, the uptake of these strategies was slow and in 2006, the WHO published a revised plan; ‘The Global Strategy for Further Reducing the Leprosy Burden and Sustaining Leprosy Control Activities 2006–2010’ [11]. This focused on ‘morbidity control’ to further promote the timely detection of new cases, their treatment with effective multidrug chemotherapy, the prevention of disability and rehabilitation. The strategy emphasised equitable resource distribution and the provision of affordable and easily accessible care. Leprosy has long been a source of stigma thus socio-educational programmes were prioritised to dispel fear and motivate individuals to seek treatment.

Leprosy is a leading communicable cause of neurological disability throughout the globe [12]. As with this gentleman, ensuring timely diagnosis and treatment is the most effective way of mitigating damage to the sensory, motor and autonomic function of peripheral nerves. The WHO definition of Grade 2 disability (G2D) has been proposed as a more robust indicator of international progress than leprosy prevalence because it is less susceptible to operational factors such as treatment duration and case-finding methodology. In 2012 Msyamboza showed that 21 % of patients in their community-based cross sectional study in Malawi had a G2D [13]. The Enhanced Global Strategy for Further Reducing the Disease Burden due to Leprosy for 2011–2015, set a target to reduce new cases of leprosy with G2D per 100 000 total population by 35 % relative to 2010. Looking forward, their aim is to reduce the burden to 1 new G2D case per one million population by 2020 [14]. However, a criticism of the indicator is that such a neurological assessment may be too complex for some developing settings, especially for clinicians that haven’t received specific training and have no means of monitoring local prevalence [15].

The WHO has defined the elimination of leprosy as a ‘national prevalence of less than 1 case per 10 000 population’ [16]. Malawi fulfils this criteria, however Msyamboza et al. showed that between 2006 and 2011, the prevalence and detection rates actually increased [11]. Furthermore, cure rates for paucibacillary (1–5 lesions) and multibacillary (>5 lesions) leprosy were 63 and 33 % respectively; substantially below the expected 80 % [13]. This gentleman was misdiagnosed at the community health centre which indicates that despite global initiatives, cases of lepromatous leprosy are still missed in this region of Malawi. Attributable factors may include a lack of decentralisation to health centres for diagnosis and treatment, insufficient knowledge and skills of health centre staff, poor registration and recording systems, and a lack of political commitment by government and health partners [13]. However, to our knowledge there are no recent studies investigating these potential issues in Malawi. In addition to global efforts, individualised, national systems of detection, contact tracing and the provision of affordable treatments are needed to definitively eradicate leprosy in these settings [17].

## Conclusions

In conclusion, beyond the optimal implementation of WHO strategies, global eradication requires the development of national initiatives that detect and treat early-stage disease. There is also a need for novel diagnostic and epidemiologic tools, more efficacious chemoprophylactic regimens and vaccine development. In Malawi, future research should delineate factors influencing the detection and management of lepromatous leprosy in the local community setting. Overall, this case illustrates the need for continued efforts that promote educational programmes for health professionals, sustain control activities in the developing world and promote investment in primary care systems.

**Table:** 

**Learning Points**
➢ Leprosy is still a prevalent communicable disease and a leading cause of long term physical disability even in countries that have statistically achieved the WHO elimination target
➢ Clinicians must consider leprosy in those with a significant contact history and be aware that leprosy can lead to severe multi-system sequelae
➢ Wider accessibility to clinical laboratories equipped with the resources to confirm leprosy could improve detection and cure rates, reduce misdiagnosis and unnecessary treatment and enhance patient follow-up.
➢ Further research is needed to develop and implement national strategies to address local factors influencing the detection and management of lepromatous disease

## Additional file

Additional file 1: Multilingual abstracts in the six official working languages of the United Nations. (PDF 373 kb)
