# Supplementary material for: A case of leprosy in Malawi. Making the final push towards eradication: a clinical and public health perspective
Source: Infect Dis Poverty. 2016 Sep 2;5(1):90. doi: 10.1186/s40249-016-0176-z (PMC5009641; doi:10.1186/s40249-016-0176-z)
Supplement: Additional file 1: — Multilingual abstracts in the six official working languages of the United Nations. (PDF 373 kb) [file 40249_2016_176_MOESM1_ESM.pdf]

Translation of the abstract into the five official working languages of the United Nations

العامة والصحة العيادي المنظور من :عليه القضاء نحو النهائية بالدفعة القيام .مالاوي في الجذام مرض قضية

ماي سارة ولوسي روث يرون

تلخيص

لكل حالة 1>) الجذام مرض على للقضاء الدولي الهدف حققت قد مالاوي أن نجد الاحصائية، الناحية من الحديث عند المؤلفان ويناقش .الطويل المدى على الجسدية للإعاقة الرئيس السبب يعتبر المرض يزال لا ولكن ،(نسمة 10.000 .العصيات متعدد ورمي بجذام مصابًا مالاوي في المقاطعة مستشفى إلى قدم سنة 39 يبلغ رجل حالة هذا حالةال تقرير في ظل في وذلك الجذام مرض عن الكشف تعيق المحلية الحواجز أن إلى أشار ما "حساسية" بوصفها مبدئيًا الحالة إدارة وأمكن الخصية بالتهاب الرجل هذا إصابة ثبتت وقد النظام متعدد مرض وهو والجذام .هذا النامي الأولية الصحية الرعاية نظام ويُعد .الطويل المدى على المرضية وتحويط المضاعفات تشخيص إمكانية من المظاهر بهذه التوعية وستزيد .الورمي لدى المرض هذا على القضاء نحو سعينا في ضرورة والمهني العام والتعليم والإدارة المرض، اكتشاف نظم تحسين الاستراتيجية" في الموضحة الأهداف تحقيق إلى نسعى أن فيجب الدولي، الصعيد وعلى .للخطر المعرضين السكان الوطني، المستوى وعلى ."2011-2015 لأعوام الجذام بسبب المرض عبء تقليل من المزيد لتحقيق المعززة العالمية وضع يسهل أن ويمكن .الجذام على القضاء تعوق التي المحلية العوامل المستقبل في الأبحاث تستهدف أن ينبغي فإنه من الموبوءة المناطق أجل من كفاءة أكثر والتطعيم الكيميائي العلاج نظم من والمزيد جديدة ووبائية تشخيصية أدوات .الجهود هذه

Translated from English version into Arabic by Fathia Sobhi, through

TRANSLATORS
WITHOUT BORDERS

马拉维麻风病 1 例：从临床和公共健康的角度为消除麻风病做出的最后努力

Cieron Roe and Lucy Sarah May

摘要

据统计，马拉维已经达到了消除麻风病的国际目标（<1 例/万人），但该疾病仍被认为是长期致残的首要原因。在这个病例报告中，作者讲述在马拉维的社区医院中一个 39 岁男患者患有多菌型麻风，即瘤型麻风。该病例最初检查确认为'过敏'，对于发展中的初级保健系统来说，检测出该麻风病例在当地是有障碍的。麻风病是一种多系统疾病，该患者证实患有瘤型麻风伴发睾丸炎。认识到存在这些症状会增加对其并发症的诊断和避免患者长期发病。在高危人群中制定检测、管理、公共及专业教育的优化系统对努力消除麻风病是必不可少的。在国际层面上，我们必须努力完成的目标是“增强全球战略来进一步降低 2011-2015 麻风病的疾病负担”。在国家层面上，未来的相关研究应该关注当地阻碍消除麻风病的因素。在麻风病流行地区开发出新的诊断和流行病学的工具，可能会促进制定出更有效的化学预防和接种方案。

Translated from English version into Chinese by Sun Lei, edited by Yang Pin, through

CHINESE CENTER FOR DISEASE CONTROL AND PREVENTION
NATIONAL INSTITUTE OF PARASITIC DISEASES

Un cas de lèpre au Malawi. Franchir la dernière étape vers l'éradication : une perspective clinique et de santé publique

Cieron Roe et Lucy Sarah May

Résumé

D'un point de vue statistique, le Malawi a réussi à atteindre l'objectif international d'élimination de la lèpre (<1 cas pour 10 000 personnes), néanmoins cette maladie continue d'être considérée comme la principale cause de handicap physique à long terme. Les auteurs rapportent ici le cas d'un homme de 39 ans qui s'est présenté à un hôpital de district au Malawi avec une lèpre lépromateuse multibacillaire. Cette affection a dans un premier temps été prise en charge à titre d'« allergie », ce qui suggère que des obstacles locaux empêchent la détection de la lèpre dans ce système de soins primaires en développement. La lèpre constitue une maladie multisystémique et cet homme présentait des signes d'orchite lépromateuse. La sensibilisation à ces manifestations améliorera le diagnostic des complications et permettra de prévenir la morbidité à long terme. L'optimisation des systèmes de détection, la formation à la prise en charge, la sensibilisation du public et la formation professionnelle sont essentielles à nos efforts d'éradication parmi ces populations à risque. Nous devons nous efforcer de satisfaire les objectifs stipulés dans le cadre de la « Stratégie mondiale renforcée visant à réduire encore la charge de morbidité due à la lèpre (2011-2015) » sur le plan international. À l'échelon national, les futures recherches doivent circonscrire les facteurs locaux qui entravent l'éradication de la lèpre. Le développement de nouveaux outils diagnostiques et épidémiologiques, de traitements chimioprophylactiques plus efficaces et la vaccination dans les régions endémiques devraient faciliter ces efforts.

Translated from English version into French by eric ragu, through

TRANSLATORS
WITHOUT BORDERS

Случай заболевания проказой в Малави. Заключительный этап на пути искоренении заболевания: клинические аспекты и вопросы охраны здоровья населения

Киран Роу и Люси Сара Мей

Краткий Обзор

Статистически, Малави достигла международных показателей по ликвидации лепры (<1 случая на 10 тысяч человек), но это заболевание до сих пор является одной из ведущих причин длительной нетрудоспособности населения. Авторы описывают историю болезни 39-летнего мужчины, поступившего в районную больницу в Малави с мультибациллярной лепроматозной проказой. Первоначально ему был проведен курс лечебных мероприятий по диагнозу «аллергия», что указывает на недостатки работы местных медицинских учреждений этой развивающейся системы первичного медико-санитарного обслуживания в диагностике лепры. Лепра является мультисистемным заболеванием, и у данного больного наблюдалась клиническая

картина лепроматозного орхита. Информированность медперсонала и понимание подобной симптоматики необходимо для успешной диагностики осложнений и снижения долгосрочной заболеваемости лепрой. Усовершенствование методов диагностики и лечения, а также повышение квалификации медицинских кадров и учебно-воспитательная работа среди населения играют ключевую роль в борьбе за искоренение лепры среди больных повышенной группы риска. На международном уровне мы должны стремиться к достижению показателей, обозначенных в «Усовершенствованной глобальной стратегии по дальнейшему снижению заболеваемости лепрой на 2011-2015 годы». На государственном уровне будущие исследования должны быть направлены на выявление местных факторов, препятствующих окончательной ликвидации заболевания лепрой. Разработка новых диагностических и эпидемиологических методик, более эффективные схемы лечения химиопрофилактическими агентами и вакцинация населения эндемичных районов будут способствовать успешной реализации этих планов.

Translated from English version into Russian by Tatiana Petrosyan, through

TRANSLATORS WITHOUT BORDERS

Un caso de lepra en Malaui. Último impulso hacia la erradicación: perspectiva clínica y de salud pública.

Cieron Roe y Lucy Sarah May

Resumen

En términos estadísticos, Malaui logró alcanzar el objetivo internacional para la eliminación de la lepra (<1 caso cada 10 000 personas). Sin embargo, todavía se considera que la enfermedad es una de las mayores causas de discapacidad física a largo plazo. En este informe, los autores discuten el caso de un hombre de 39 años que se presentó en un hospital de distrito en Malaui con lepra lepromatosa multibacilar. En un primer momento, se trató a la enfermedad como “alergia”, lo que sugiere que en este sistema de salud primario en desarrollo existen barreras locales que obstaculizan la detección de la lepra. La lepra es una enfermedad multisistémica, y este hombre presentaba evidencias de orquitis lepromatosa. Tomar conciencia de estas manifestaciones mejorará el diagnóstico de las complicaciones y evitará la morbilidad a largo plazo. La mejora de los sistemas de detección, la administración y la educación pública y profesional son de vital importancia para nuestros esfuerzos de erradicar la enfermedad en estas poblaciones de riesgo. En el ámbito internacional, debemos luchar para lograr los objetivos delineados en la “Estrategia global mejorada para reducir la carga de morbilidad debida a la lepra: 2011 - 2015”. En el ámbito nacional, la investigación que se lleve a cabo en el futuro debería encargarse de delinear cuáles son factores locales que impiden la erradicación de la lepra. El desarrollo de nuevas herramientas epidememiológicas y de diagnóstico, la mejora de la eficiencia de los régimes quimoprofilácticos y la vacunación en las regiones endémicas podrían facilitar estos esfuerzos.

Translated from English version into Spanish by Mpgorgone, through

TRANSLATORS WITHOUT BORDERS
